# 

**Published:** 1905-08

**Authors:** 


					﻿Ipixty-first Year.
Vol. LXI.	AUGUST, 1905.	No. 1 '
BUFFALO i
MEDICALJOURNAL
- -	1845 by AUSTIN FLINyf'Mt p.r* ,
•*« < i * »X
!~.-;.CL. .rnXr.5.jrr!C£ I -
| EDITOR:	V
"'ll I th OWILLIAM WARREN POTTER, M. DxM Q Q, >
ASSISTANT EDITORS:
WILLIAM C. KRAUsj, M. D.	NELSON W. WILSON, M. D.
ASSOCIATE EDITORS:
JAMES WRIGHT PUTNAM, M. D. ERNEST WENDE, M. D.
JOHN PARMENTER, M. D.	JOHN A. MILLER, Ph. D
HARVEY R. GAYLORD, M. D. MAUD J. FRYE, M. D.
				

